# Refractory Hypercalcemia of Malignancy in Squamous Cell Carcinoma of the Buccal Mucosa With Skeletal Muscle Metastasis

**DOI:** 10.7759/cureus.71816

**Published:** 2024-10-18

**Authors:** Sai Krishna Reddy Bana, Jagannath S Dhadwad, Kunal Modi, Kumar Roushan, Prabhanjan Kulkarni, Chandan Dash

**Affiliations:** 1 Internal Medicine, Dr. D. Y. Patil Medical College, Hospital and Research Centre, Dr. D. Y. Patil Vidyapeeth (Deemed to be University), Pune, IND

**Keywords:** buccal cancer, hypercalcemia, hypercalcemic aki, skeletal muscle metastasis, squamous cells carcinoma

## Abstract

Refractory hypercalcemia of malignancy (RHOM) is a challenging and often life-threatening condition characterized by persistently high serum calcium levels despite standard treatments. It is commonly associated with malignancies such as squamous cell carcinoma (SCC) of the lung, breast cancer, and multiple myeloma. However, studies on head and neck cancers, including SCC of the oral cavity, suggest that hypercalcemia can occur but is relatively rare. We report a case of a 45-year-old male with SCC of the buccal mucosa who presented with severe, refractory hypercalcemia. Despite aggressive hydration, bisphosphonates, calcitonin therapy, and glucocorticoids, serum calcium levels remained elevated. The patient was subsequently treated with hemodialysis, but despite this intervention, his clinical status did not improve, ultimately leading to his mortality. This case highlights the challenges in managing RHOM and underscores the need for better therapeutic strategies. Timely recognition and innovative treatment approaches are crucial for improving patient outcomes in refractory cases of hypercalcemia of malignancy.

## Introduction

Hypercalcemia is a serious electrolyte disturbance often associated with malignancies, including squamous cell carcinoma (SCC). While hypercalcemia is well-documented in certain cancers such as lung and breast cancers, its incidence in SCC of the buccal mucosa is less frequently studied [1]. The management of refractory hypercalcemia of malignancy (RHOM) involves a multifaceted approach due to its resistance to standard treatments. Initial management approaches include aggressive intravenous hydration and loop diuretics [2]. Calcitonin has a limited role; it exerts its anti-hypercalcemia effects by reducing bone resorption but cannot be continued for more than 48-72 hours due to tachyphylaxis [3]. Bisphosphonates and denosumab are potent drugs used in specific situations like in those with hypercalcemia of malignancy or renal failure, respectively. Unlike liver, lung, and brain metastases seen in SCC of the head and neck, isolated skeletal muscle involvement is very rare [4]. Our case report discusses a rare metastatic involvement of the adductor magnus muscle due to primary SCC of the head and neck. This case report addresses the significant challenges encountered in managing RHOM despite implementing standard treatment protocols. We faced difficulties in controlling elevated calcium levels with conventional therapies. The case highlights the need for advanced therapeutic approaches and the importance of individualized treatment plans for the effective management of RHOM.

## Case presentation

**Figure 1:**
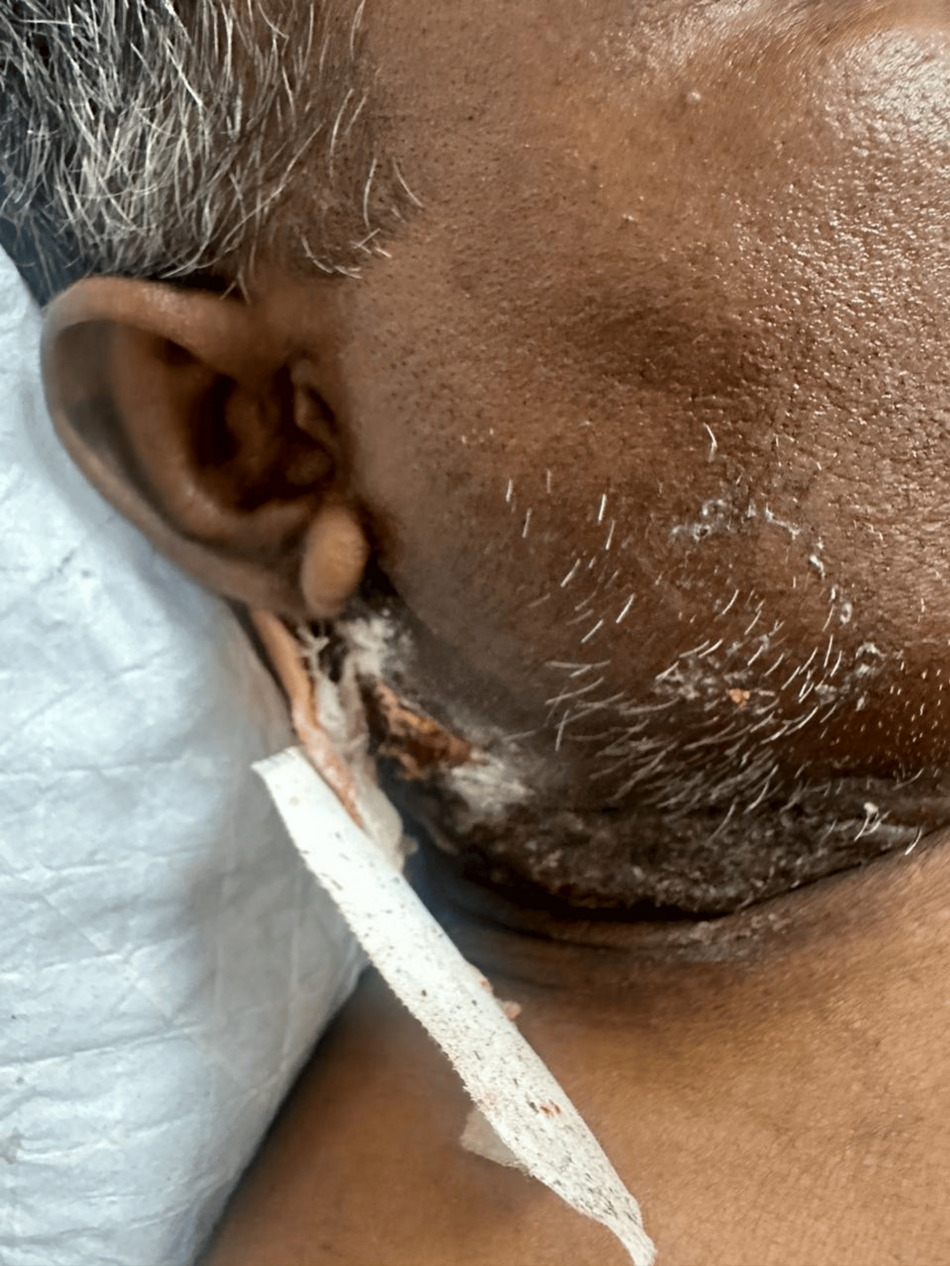
Pus discharge from the operated site

A 45-year-old male with SCC of the buccal mucosa, for which he had undergone right wide local excision of the tumour along with modified radical neck dissection with right maxillectomy followed by buccal fat pad reconstruction surgery a month prior to this admission, presented with complaints of swelling and pus discharge from the operated site (Figure 1).

He also complained of pain in the right thigh for the past week. An examination of the right thigh revealed a palpable, soft mass with severe tenderness over the posterior portion. Ultrasonography of the right thigh showed a hypoechoic solid-cystic mass lesion measuring 7.2 x 20 x 5.6 cm with mild internal vascularity in the deep intramuscular compartment adjacent to the posterior aspect of the femur cortex. Medical oncology was consulted, and both an MRI of the right thigh and a whole-body PET-CT were advised.

**Figure 2:**
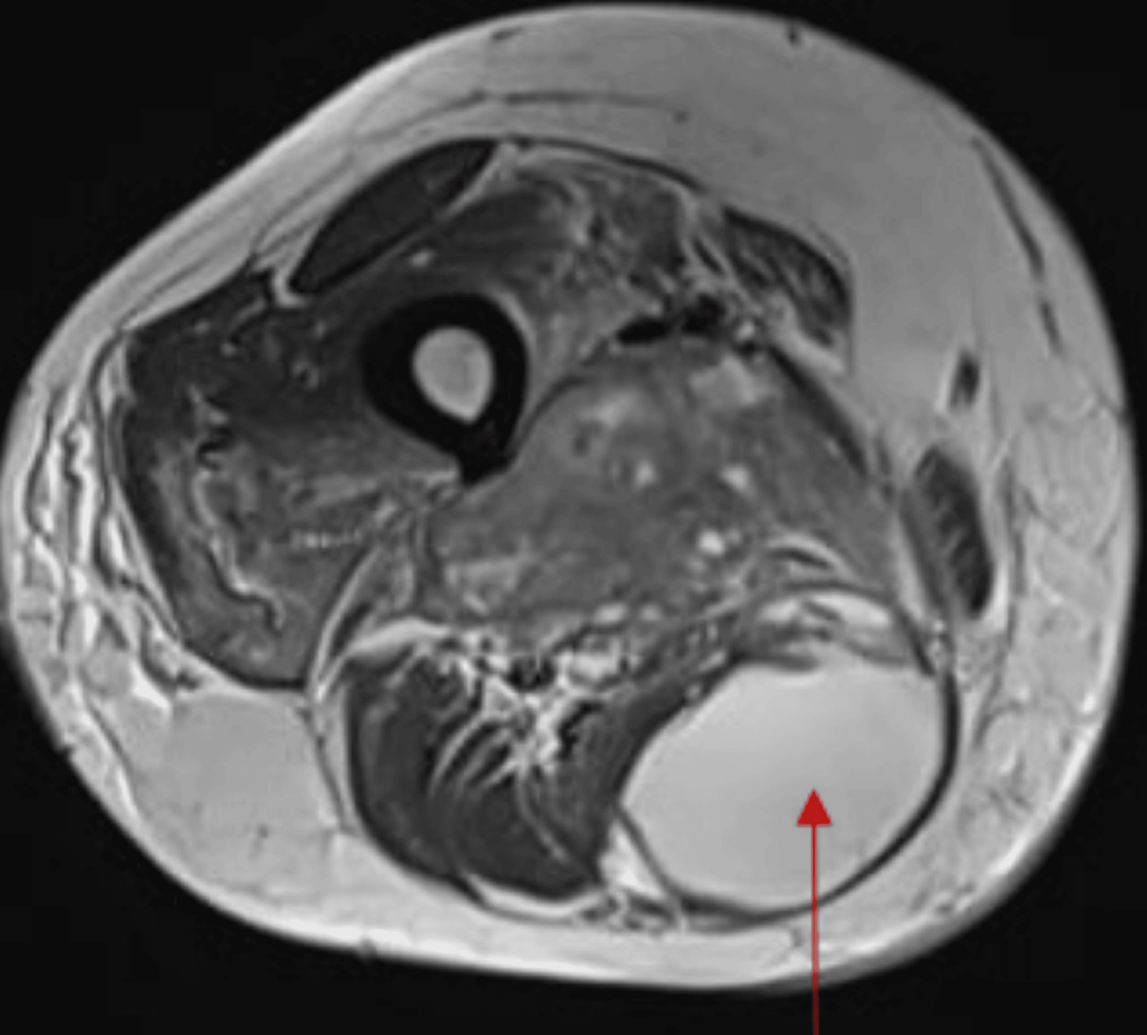
MRI of the right thigh T2-weighted image showing hyperintense collection (red arrow)

**Figure 3:**
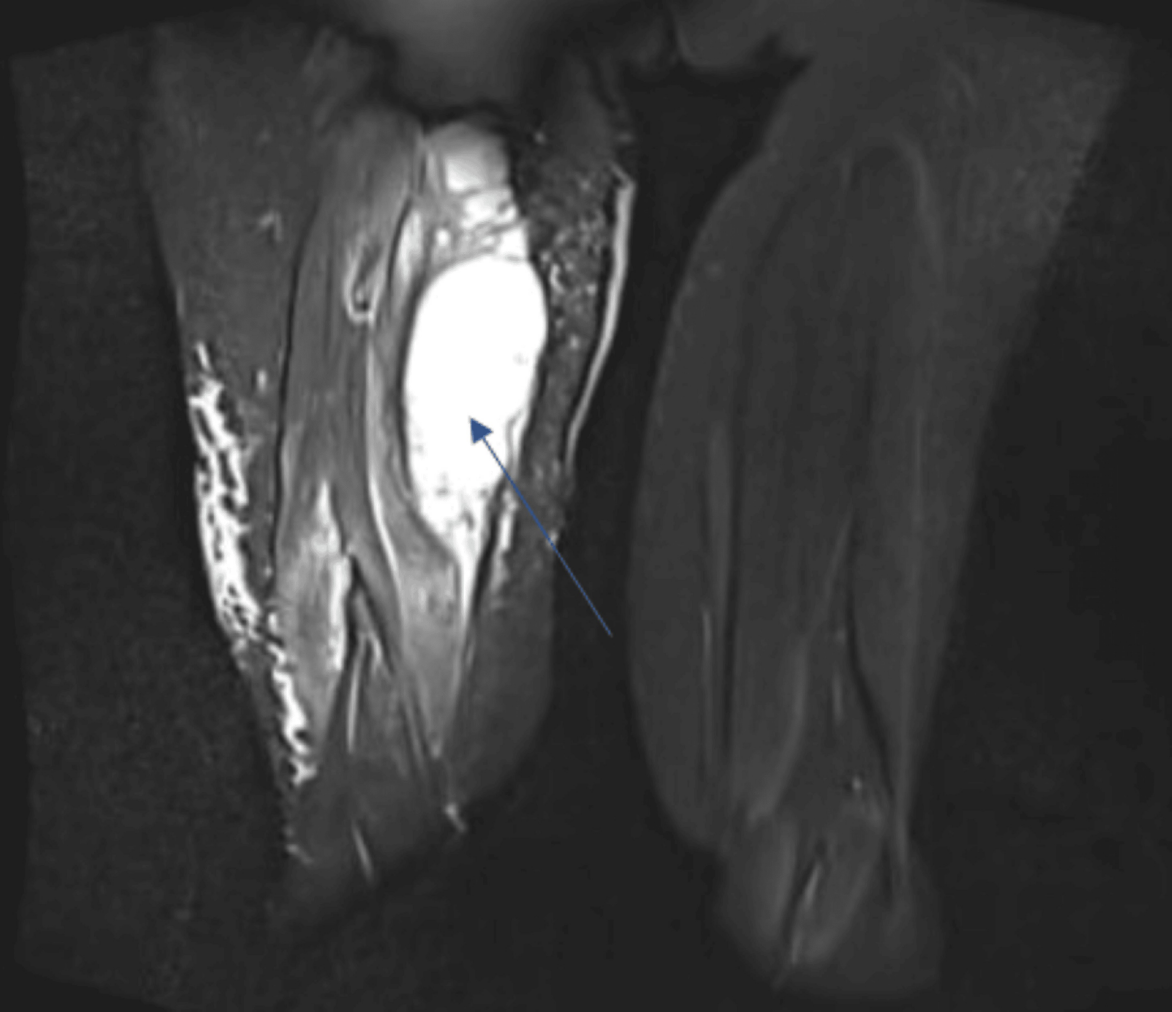
MRI of the right thigh in coronal section (stir sequence) showing hyperintensity (blue arrow)

The MRI reported a large, ill-defined, lobulated solid-cystic heterogeneous enhancing soft tissue lesion in the posteromedial compartment of the right thigh, predominantly involving the adductor magnus muscle and adjacent muscle planes and fat. The lesion had arterial feeders from the right profunda femoris artery and completely encased the sciatic nerve. Multiple cystic areas and fluid levels within the lesion were consistent with a malignant mass, likely metastasized from oral carcinoma (Figures 2, 3).

Fine needle aspiration cytology (FNAC) of the thigh mass confirmed squamous cell metastasis. Upon admission, routine blood investigations were conducted, and their course during the hospital stay is summarized in Table 1.

**Table 1:** Blood parameters Hb: haemoglobin; TLC: total leucocyte count; PTH: parathyroid hormone; g/dL: grams per decilitre; microL: microlitre; mg/dL: milligrams per decilitre; mmol/L: millimoles per litre; pg/mL: picograms per millilitre; N/A: not available

Lab parameters (normal reference range)	DAY 1	DAY 3	DAY 5	DAY 7	DAY 9	DAY 11	DAY 13	DAY 15
Hb (13.2 – 16.6 g/dL)	12 g/dl	11.4 g/dL	10.3 g/dL	10.4 g/dL	10.9 g/dL	10.7 g/dL	10.4 g/dL	11.3 g/dL
TLC (4000-1000/microL)	27750/microL	21990/microL	21200/microL	22100/microL	23000/microL	24000/microL	23000/microL	32200/microL
Platelets (150000-410000/microL)	447000/microL	382000/microL	325000/microL	337000/microL	361000/microL	298000/microL	231000/microL	180000/microL
Urea (17- 49 mg/dL)	71 mg/dL	57 mg/dL	40 mg/dL	60 mg/dL	43 mg/dL	67 mg/dL	83 mg/dL	78 mg/dL
Creatinine (0.6 -1.35 mg/dL)	2.52 mg/dL	1.89 mg/dL	1.3 mg/dL	1.81 mg/dL	1.30 mg/dL	1.2 mg/dL	1.5 mg/dL	1.27 mg/dL
Sodium (136 – 145 mmol/L)	122 mmol/L	123 mmol/L	127 mmol/L	128 mmol/L	129 mmol/L	141 mmol/L	151 mmol/L	143 mmol/L
Calcium (8.6 – 10.2 mg/dL)	15.5 mg/dL	14.7 mg/dL	13.5 mg/dL	13 mg/dL	12.1 mg/dL	17.4 mg/dL	17.6 mg/dL	22.3 mg/dL
Phosphorus (2.6-4.7 mg/dL)	4.8 mg/dL	N/A	N/A	N/A	N/A	3.7 mg/dL	N/A	N/A
PTH (15-65 pg/mL)	3.3 pg/mL	N/A	N/A	N/A	N/A	N/A	5.7 pg/mL	N/A
Serum albumin (3.5-5.2 g/dL)	3.7 g/dL	N/A	N/A	N/A	N/A	N/A	N/A	N/A

**Figure 4:**
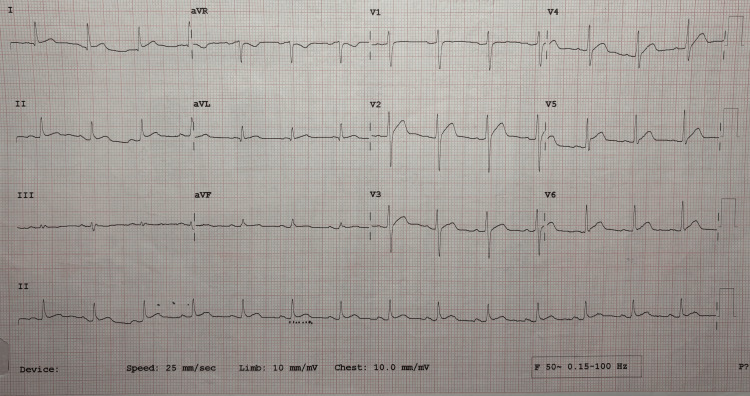
ECG showing QTc shortening due to elevated serum calcium

Elevated serum calcium levels were confirmed by repeat testing. Serum PTH was found to be low, indicating humoral hypercalcemia of malignancy (HHM) secondary to SCC of the buccal mucosa. The patient also developed acute kidney injury (AKI) secondary to elevated calcium. Hypercalcemia was addressed promptly with aggressive hydration with intravenous normal saline given as a one-litre bolus and later at a rate of 125 ml/hour, followed by injection of furosemide at 40 mg intravenous twice daily dosage. Calcitonin was initiated at a dose of 4 international units per kilogram subcutaneously every 12 hours on day 1 and increased to 8 International Units per kilogram subcutaneously every 6 hours on day 2. After 72 hours, calcitonin was stopped due to tachyphylaxis. Despite these interventions, calcium levels remained persistently elevated. Zoledronic acid (4 mg) was administered intravenously as a single dose, but calcium levels continued to exceed the normal range. Given the RHOM, a trial of steroids was also initiated. Continuous ECG monitoring revealed QT shortening (Figure 4).

The patient also experienced severe abdominal pain and constipation due to the extremely high calcium levels. A nephrology consult was obtained, and urgent hemodialysis was scheduled with a low-calcium dialysate. He underwent two hemodialysis sessions. However, serum calcium levels remained significantly elevated. On day 15 of admission, with serum calcium levels at 22.3 mg/dl, the patient experienced a cardiac arrhythmia followed by cardiac arrest and could not be resuscitated.

## Discussion

Hypercalcemia of malignancy can be classified into two types based on its cause: one due to bone metastasis, resulting in local osteolysis, and the second due to the secretion of hypercalcemic factors by solid tumours, known as HHM. Hypercalcemia of malignancy occurs in 2 to 30 percent of cancer patients and is linked to significant morbidity and mortality [3]. Several hormonal factors, like ectopic secretion of parathyroid hormone by tumours and prostaglandins activating osteoclasts, along with non-hormonal hypercalcemic factors, contribute to malignant hypercalcemia [5]. Parathyroid hormone-related peptide (PTH-rP) is one of the commonest and most potent contributors to HHM, secreted by most solid epithelial tumours [6,7].

The incidence of hypercalcemia in patients with SCC of the oral cavity is 4.1%, increasing to 40% among those in the terminal stage of the disease [8]. Initial tests for evaluating hypercalcemia should include PTH levels to differentiate between PTH-related and non-PTH-related hypercalcemia. PTH-related hypercalcemia is often due to primary hyperparathyroidism, while non-PTH-related hypercalcemia can result from malignancies, granulomatous diseases, endocrine disorders, or vitamin D intoxication [9].

Hypercalcemia is clinically significant due to its association with symptomatic worsening and increased mortality. It can lead to various complications like central nervous system depression, muscle weakness, cardiac abnormalities, gastrointestinal disturbances, or renal failure, depending on its severity and rate of onset [10]. In our case, the patient experienced cardiac complications in the form of QT shortening and arrhythmia, which led to sudden cardiac arrest.

Patients with SCC experiencing their first episode of hypercalcemia of malignancy have a short overall survival, with a median of 64 days. Independent negative prognostic factors include brain metastases, a calcium level of 12 mg/dl, and hypoalbuminemia [11].

For symptomatic patients with severe hypercalcemia, initial treatment includes intravenous normal saline (NS), calcitonin, and bisphosphonates. NS promotes calcium excretion and should be administered at 200-300 mL/hr. Calcitonin acts quickly but temporarily, while bisphosphonates, such as zoledronic acid or pamidronate, reduce calcium levels over a few days. Calcitonin's effectiveness is typically limited to the first 48 hours due to tachyphylaxis [12]. Bisphosphonates are particularly useful for malignancy-related hypercalcemia and can prevent bone complications [13]. Serum creatinine must be monitored before administering further doses of zoledronic acid. Dose adjustments according to creatinine clearance are as follows: 4 mg for glomerular filtration rate (GFR) > 60 mL/min, 3.5 mg for GFR: 50-60 mL/min, 3.3 mg for GFR: 40-49 mL/min, and 3.0 mg for GFR: 30-39 mL/min. Osteonecrosis of the jaw is a potential complication of bisphosphonate use [13]. Denosumab, which inhibits RANKL, is an alternative for patients unresponsive to bisphosphonates or with kidney impairment [14].

Gallium nitrate acts by inhibiting calcium absorption from bone [15]. Cinacalcet is preferred for hemodialysis patients and parathyroid cancer-related hypercalcemia [16]. If other treatments fail, hemodialysis may be required, especially in cases of severe heart or renal failure [17]. A dialysate with calcium-free acetate solution or one with the lowest calcium level should be used. 

Glucocorticoids are useful in conditions with elevated 1,25-dihydroxy vitamin D, such as lymphomas or granulomatous diseases. Increased calcitriol in patients with solid tumours is more common than previously reported and does not respond well to antiresorptive therapy [18]. Contrary to this statement, our patient's vitamin D levels were lower than normal at 17 ng/ml despite which resistance to anti-resorptive therapy was noted. In our case, corticosteroids were used without optimal clinical response.

In our patient, all available treatment options were attempted but failed to achieve a desirable response. Currently, newer drugs targeting PTH-rP are in development and may offer potential solutions for cases refractory to existing treatment modalities [19].

Skeletal muscle metastasis (SMM) is uncommon and challenging to detect with standard ultrasound, MRI, or CT, especially in patients who do not have a known history of cancer [20]. Our case involved skeletal metastasis in the form of a right thigh soft tissue mass, a very rare presentation [4]. Metastasis to the adductor magnus muscle was confirmed by FNAC. The metastasis was further complicated by metabolic derangements not responding to standard therapy which posed significant challenges in the management.

## Conclusions

HHM in oral SCC is a severe prognostic indicator. Antihypercalcemic therapy plays a crucial palliative role, temporarily alleviating symptoms and reducing discomfort in the terminal stage of illness. RHOM remains an underexplored issue with a significant need for effective treatments. Addressing the underlying pathophysiological mechanisms through antiresorptive therapy, glucocorticoids, phosphorus, and possibly cinacalcet may not be beneficial in a few cases. New treatments for refractory hypercalcemia are eagerly anticipated.
